# Identifying Early Inflammatory Changes in Monocyte-Derived Macrophages from a Population with IQ-Discrepant Episodic Memory

**DOI:** 10.1371/journal.pone.0063194

**Published:** 2013-05-06

**Authors:** Eric J. Downer, Raasay S. Jones, Claire L. McDonald, Eleonora Greco, Sabina Brennan, Thomas J. Connor, Ian H. Robertson, Marina A. Lynch

**Affiliations:** 1 Trinity College Institute of Neuroscience and Physiology Department, Trinity College, Dublin, Ireland; 2 Trinity College Institute of Neuroscience and School of Psychology, Trinity College, Dublin, Ireland; Universidad Pablo de Olavide, Centro Andaluz de Biología del Desarrollo-CSIC, Spain

## Abstract

**Background:**

Cells of the innate immune system including monocytes and macrophages are the first line of defence against infections and are critical regulators of the inflammatory response. These cells express toll-like receptors (TLRs), innate immune receptors which govern tailored inflammatory gene expression patterns. Monocytes, which produce pro-inflammatory mediators, are readily recruited to the central nervous system (CNS) in neurodegenerative diseases.

**Methods:**

This study explored the expression of receptors (CD11b, TLR2 and TLR4) on circulating monocyte-derived macrophages (MDMs) and peripheral blood mononuclear cells (PBMCs) isolated from healthy elderly adults who we classified as either IQ memory-consistent (high-performing, HP) or IQ memory-discrepant (low-performing, LP).

**Results:**

The expression of CD11b, TLR4 and TLR2 was increased in MDMs from the LP group when compared to HP cohort. MDMs from both groups responded robustly to treatment with the TLR4 activator, lipopolysaccharide (LPS), in terms of cytokine production. Significantly, MDMs from the LP group displayed hypersensitivity to LPS exposure.

**Interpretation:**

Overall these findings define differential receptor expression and cytokine profiles that occur in MDMs derived from a cohort of IQ memory-discrepant individuals. These changes are indicative of inflammation and may be involved in the prodromal processes leading to the development of neurodegenerative disease.

## Introduction

Neuroinflammatory changes develop with age and are a feature of most neurodegenerative diseases including Alzheimer’s disease (AD). Epidemiological studies indicate that long-term non-steroidal anti-inflammatory treatment reduces the risk of developing AD [1] suggesting that inflammatory processes may contribute to very early changes in the pathogenesis of the disease. The inflammatory changes in the central nervous system (CNS) in normal ageing and AD are characterised by microglial activation with resultant changes in microglial phagocytic activity and neurotoxic consequences [2]–[4]; the evidence indicates that some of these CNS changes have systemic parallels. The number of blood-derived monocytes is increased in AD patients [5], and it has been reported that circulating monocytes express specific cell adhesion molecules (CAMs) that are altered with age, and in patients with AD and Mild Cognitive Impairment (MCI) [6], [7]. Interestingly, circulating monocytes readily infiltrate the brain in murine models of AD [8], [9] and evidence indicates that blood-borne monocytes can act as amyloid phagocytes and may be involved in amyloid-β (Aβ) clearance [10]. These findings, alongside others which show that monocytes, in addition to *in vitro* differentiated macrophages, express several AD-related genes [11], suggest that circulating monocytes may be a valuable model system in assessing the role of microglial cells in the pathogenesis of neurodegenerative conditions.

Human monocytes express an array of CAMs and receptors that are crucial for cell-cell and cell-matrix interactions, transmigration and innate immune responses. Of these, CD11b is a receptor for intercellular adhesion and is a phenotypic monocytic cell marker, the expression of which increases with age, indicating a more activated phenotype [6]. Monocytes/macrophages also express a diverse array of toll-like receptors (TLRs), a family of signalling pattern recognition receptors that mediate innate immunity by recognising conserved motifs of microbial origin known as pathogen-associated molecular patterns (PAMPs) and endogenous damage-associated molecular patterns (DAMPs) that are released by injured tissue [12]. Human blood monocytes express TLR1, TLR2, TLR4, TLR5, TLR6, TLR8, and TLR9 mRNA, with TLR2 and TLR4 being the most highly expressed [13]. TLRs continue to emerge as key players in CNS diseases and much data link TLRs with AD pathology, with polymorphism in the *tlr2* and *tlr4* gene linked with the disease [14]. The classical monocyte marker, CD14, a glycosylphosphatidyl inositol (GPI)-anchored myeloid glycoprotein, acts as a co-receptor for TLRs and is responsible for the uptake of Gram-negative bacterial lipopolysaccharide (LPS) via macropinocytosis [15]. Indeed, TLR4 acts as the primary signalling receptor for LPS [16], with TLR2 further required for LPS-induced TLR4 signalling [17].

In an attempt to quantify relative decline and explore discrepancies between targeted cognitive domains that are central to ageing (e.g. episodic memory) and a proxy measure of lifetime cognitive capacity, we have identified a group of otherwise healthy adults who we classify as low performers (LP) because their memory performance is discrepant with their estimated IQ. The advantage of adopting such an approach is threefold. First, it allows quantification of true age-related changes in the strength or weakness of memory relative to an estimate of lifetime cognitive capacity. Second, it allows one to dissociate domain-specific changes in memory function. Third, it allows classification of individuals as high or low memory performers based on standardised measures of memory relative to IQ that can be utilised, not only in research, but also in a clinical setting. The purpose of the study was to compare the expression of receptors (CD11b, TLR2 and TLR4) on circulating monocyte-derived macrophages (MDMs) and the response of these cells to LPS in samples prepared from the LP cohort and a cohort which we classified as IQ memory-consistent (high-performing, HP) individuals. We show that MDMs isolated from the LP group display enhanced expression of CD11b, TLR2 and TLR4, compared to the HP group. We further show that treatment of MDMs with LPS promoted pro-inflammatory cytokine production in HP and LP groups, but that the effect was exacerbated in MDMs prepared from the LP group.

## Methods

### Participants

Adults (35 female, 12 male) with a mean age of 71.5 years (range of 65 to 82) were recruited from the older adult participant panel of the Trinity College Institute of Neuroscience, Dublin, Ireland. Participants were assigned to low and high performing sub-groups (LP and HP respectively) based on their memory performance relative to an estimate of their intelligence. Z-scores were used to relate their performance on the Wechsler Memory Scale story recall test [WMS III UK; 18] to their scores on The National Adult Reading Test [NART; 19]. Participants were defined as LP if they scored more than 0.75 standard deviations below their NART-estimated IQ on the WMS. All other participants were classified as HP. This approach yielded 35 HPs (26 female, 9 male) with a mean age of 71.9 (SD = 4.7) and 12 LPs (9 female, 3 male) with a mean age of 71.1 (SD = 4.8). No significant difference was observed in Mini Mental State Exam (MMSE) scores between the two groups (Table 1).

**Table 1:** Demographic of subjects.

	HP	LP
N	35	12
Age (years; mean ± SD)	71.9±4.7	71.1±4.8
Sex (F/M)	26/9	9/3
MMSE	27.4±2.8	26.9±2.6
Premorbid IQ	1.13±0.37	1.20±0.29
Delayed verbal memory	1.43±0.82	−0.06±0.72*

Premorbid IQ was measured by the National Adult Reading Test (NART). Delayed verbal memory was determined by the Logical Memory subtest of the Wechsler Memory Scale.

* HP *versus* LP; memory performance was less than 0.75 z-score below their estimated premorbid IQ.

Participants received reimbursement of travel expenses to the maximum value of €20. This study was approved by the Ethics Committee of the School of Psychology at Trinity College Dublin, and all participants provided informed consent. All participants completed a detailed questionnaire about their own health and current medications, as well as any relevant health issues in their family. Participants who had a history of head injury, stroke, epilepsy, neurological conditions, major psychiatric disorder, heart attack or diabetes were excluded from the study.

### Neuropsychological Assessment

A battery of tests was administered in one session, and focused on memory and executive function. The MMSE was used as a screening measure. The NART was used as a proxy measure of general intellectual status. Memory was assessed using 3 subtests of Wechsler Memory Scale: Logical Memory I and II Verbal Paired Associates I and II Visual Reproduction I and II.

### Preparation of MDMs

Human PBMCs were prepared from heparinised venous whole blood samples (50 ml per donor) from HP and LP individuals by density separation over Lymphoprep™ (Axis-Shield, Norway). Plasma samples were separated following centrifugation, aliquoted and stored at –80°C. CD14^+^ monocytes were isolated from PBMCs using positive selection with MACS microbeads (Miltenyi Biotec, Germany) and an autoMACS cell sorting instrument using a method which results in up to 95% purity as estimated by flow cytometry [20]. The mean percentage monocytes in PBMCs from HP and LP groups was 20.5±1.0 and 18.48±1.7, respectively (*P* = 0.289; 2-tailed unpaired *t*-test). In this study, CD14^+^ cells, obtained from PBMCs following MACS sorting, were seeded (6×10^5^ cells/ml) on 24-well plates and cultured in RPMI supplemented with FBS (10%), antibiotics and human granulocyte macrophage colony-stimulating factor (10 ng/ml; R&D Systems, UK) for 7 days. At the end of this period, cells were assessed again by flow cytometry; 90.2% of the cells were CD14^+^ (Figure S1), which is consistent with previous studies that have been shown to yield >85–92% CD14^+^ cells [20]–[22]. We refer to these cells as MDMs in the present study.

### Flow Cytometry

Freshly-isolated PBMCs and MDMs were washed 3 times in FACS buffer (2% FBS, 0.1% NaN_3_ in PBS) and blocked for 20 min at 4°C with purified human IgG (1 mg/ml in FACS buffer; Sigma, UK). Cells were incubated with anti-human CD11b-APC (clone ICRF44), anti-human TLR2-FITC (clone T2.5) and anti-human TLR4-PE-Cy7 (clone HTA125) (all at 1∶100 in FACS buffer; Biosciences, UK). A minimum of 300,000 events were collected and the percentage positive staining for each cellular target was calculated. Total viable PBMCs were initially gated via forward and side scatter (demonstrated in Figure S2) and doublets were excluded via forward scatter versus pulse width signal. CD14^+^ monocytes, isolated from PBMCs using positive selection with MACS microbeads (85–92% purity [22]), were differentiated to MDMs over 7 days, and viable MDMs (90.2% CD14^+^ cells) gated via forward and side scatter. Flow cytometric analysis of CD11b^+^, TLR2^+^ and TLR4^+^ cells in PBMCs and MDMs was performed on a DAKO CyAN ADP 7 colour flow cytometer (DAKO Cytomation, UK) with Summit v4.3 software and analysed with FloJo v7.6.5 software, with gating set on appropriate type matched IgG_1_ κ isotype controls and fluorescence-minus one (FMO) controls prepared from LP and HP cohorts. Non-specific staining was further minimised by incubating cells from HP and LP groups in purified human IgG. Instrument compensation and cell subset gates were set based on unstained, isotype and FMO controls, which were all based on viable forward versus side scatter gates.

### Real-time PCR

MDMs were stimulated with LPS (100 ng/ml) and after 24 h RNA was extracted using a NucleoSpin® RNAII isolation kit (Macherey-Nagel Inc., Germany). The concentration of LPS used is in line with those used in various inflammatory paradigms in human monocytes and macrophages [23]–[25]. The concentration of RNA was determined using a UV/Vis spectrophotometer (Beckman Coulter Inc., Ireland). cDNA synthesis was performed on 1 µg RNA using a High Capacity cDNA RT Kit (Applied Biosystems, USA) according to the manufacturer’s instructions. Equal amounts of cDNA were used for RT-PCR amplification. Real-time PCR primers were delivered as “Taqman® Gene Expression Assays” containing forward and reverse primers, and a FAM-labeled MGB Taqman probe for each gene (Applied Biosystems, USA). Primers used were as follows: CD11b, TLR2 and TLR4 (Taqman® Gene Expression Assay no. Hs00355885_m1, Hs00610101_m1 and Hs00152937_m1, respectively). A 1 in 4 dilution of cDNA was prepared and real-time PCR performed using an Applied Biosystems 7300 Real-time PCR System. cDNA was mixed with qPCR™ Mastermix Plus (Applied Biosystems, USA) and the respective gene assay in a 25 µl volume (10 µl of diluted cDNA, 12.5 µl Taqman® Universal PCR Mastermix, 1.25 µl target primer and 1.25 µl GAPDH). Human GAPDH was used as an endogenous control and expression was conducted using a gene expression assay containing forward and reverse primers and a VIC-labeled MGB Taqman probe (#4326317E; Applied Biosystems, USA). Samples were run in duplicate and 40 cycles were run as follows: 10 min at 95°C and for each cycle, 15 s at 95°C and 1 min at 60°C. Gene expression was calculated relative to the endogenous control and analysis was performed using the 2^−ΔΔCT^ method. In all experiments no change in relative GAPDH mRNA expression between treatment groups was observed.

### Cytokine Analysis in Culture Supernatants

MDMs were stimulated with LPS (100 ng/ml; 24 h) and supernatants were assessed for tumor necrosis factor (TNF)α, IL-6, IL-10 and IL-12p70 production using a human pro-inflammatory-7-plex assay (MesoScale Discovery, USA) according to manufacturer’s instructions. Briefly, plates were blocked in Diluent 2 for 30 min at room temperature and duplicate samples and standards (0–10,000 pg/ml) added for 2 h at room temperature with vigorous shaking. Detection antibody solution was added for 2 h at room temperature with vigorous shaking. Plates were washed again and read buffer was added to the plate. The plate was read immediately using a Mesoscale Sector Imager plate reader and pro-inflammatory cytokine concentrations in test samples were evaluated with reference to the standard curve prepared using the 7-plex calibrator blend.

### C-reactive Protein (CRP) Measurement in Plasma

Plasma samples from HP and LP groups were analysed for concentrations of CRP by ELISA (Duoset, R&D Systems, UK) according to manufacturer’s instructions.

### Statistical Analysis

Data were analysed using a Student’s *t*-test for independent means or two-way analysis of variance (ANOVA) as appropriate. When analysis by ANOVA indicated significance (*P*<0.05), the post hoc Student Newman–Keuls test was used. Data are expressed as means ± standard errors of the mean (SEM).

## Results

### Human MDMs Isolated from the LP Group Display Enhanced Expression of CD11b, TLR2 and TLR4, Compared to HPs

**Figure 1:**
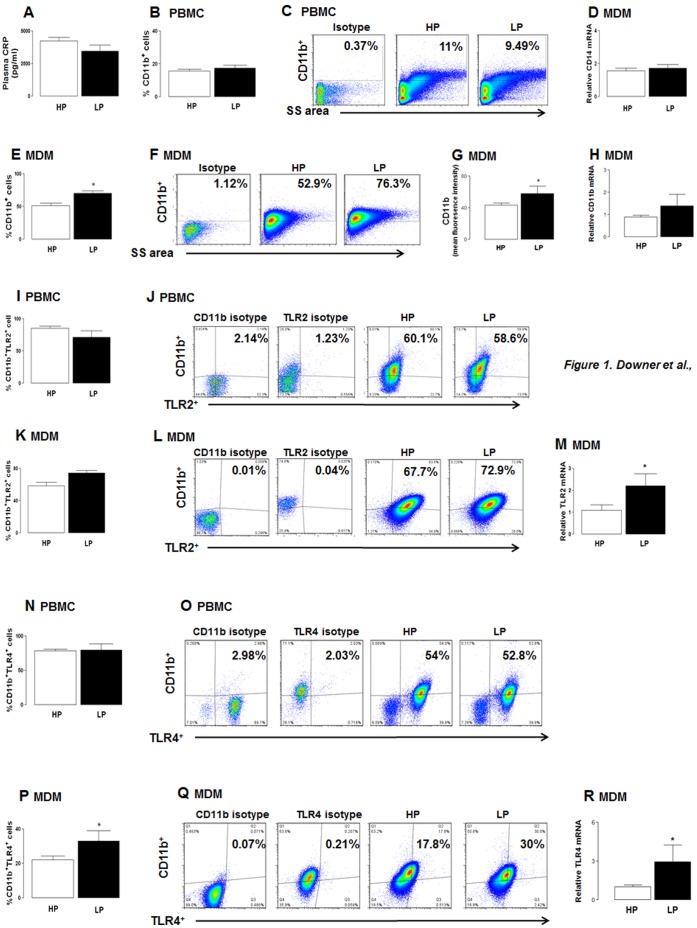
Enhanced CD11b, TLR2 and TLR4 expression in MDMs from LPs. A) Plasma isolated from HP and LP groups displayed comparable levels of CRP. Data are presented as mean ± SEM and represent triplicate determinations from 35 and 12 samples per HP and LP groups, respectively. B) CD11b expression on PBMCs was unchanged between HP and LP subjects. C) Representative plots of CD11b^+^ cells in PBMC populations. Numbers beside gated areas indicate the percentage positive cells in that area. D) CD14 expression in MDMs was similar in LP and HP individuals following 7 days in culture. E) Percentage CD11b expression was increased in MDMs derived from the LP group, compared with the HP group (*P*<0.05). F) Representative dot plots of CD11b^+^ cells in MDMs (with values beside gated areas indicating the percentage positive cells in that area) show marked differences between cohorts. G) CD11b mean fluorescence intensity on MDMs was increased in the LP group compared with the HP group (*P*<0.05). H) CD11b mRNA in MDMs derived from the LP group was slightly, though not significantly, greater than values from the HP group (*P* = 0.19). I) TLR2 expression on CD11b^+^ PBMCs was similar in HP and LP groups and this is also shown in the representative dot plots of TLR2^+^ cells (J) which indicate the percentage positive cells. K) TLR2 expression on CD11b^+^ MDMs was increased in the LP group, compared to the HP group (*P* = 0.056) and this is also shown in the representative dot plots of TLR2^+^ cells (L) which indicate the percentage positive cells. M) TLR2 mRNA expression was increased on MDMs derived from the LP group compared with the HP group (*P*<0.05). N) TLR4 expression on CD11b^+^ PBMCs was similar in HP and LP groups and this is also shown in the representative dot plots of TLR4^+^ cells (O) which indicate the percentage positive cells. P) TLR4 expression on CD11b^+^ MDMs was increased in the LP group compared with the HP group (*P*<0.05) and this is also shown in the representative dot plots of TLR4^+^ cells (Q) which indicate the percentage positive cells. R) TLR4 mRNA expression on MDMs was increased in the LP group compared with the HP group (*P*<0.05). Data are expressed as (B, E, I, K, N, P) mean percentage or fluoresence (G) expression ± SEM, or (D, H, M, R) mean relative expression ± SEM, of duplicate determinations in 35 and 12 samples per HP and LP groups, respectively.

Since neurological symptoms are often exacerbated by infection [26], we initially assessed plasma levels of the inflammatory reactant CRP in plasma from HP and LP groups. No significant difference in plasma CRP concentration was found between groups (Figure 1A). PBMCs predominantly constitute lymphocytes and monocytes, with lower numbers of natural killer (NK) and dendritic cells. CD11b is an adhesion molecule primarily expressed on the surface of monocytes, but also on activated lymphocytes and a subset of NK cells, where it mediates leukocyte adhesion and migration to orchestrate an inflammatory response [27]. Indeed, CD11b expression is upregulated by many inflammatory mediators including cytokines [28]. CD11b expression was unchanged on PBMCs isolated from the HP and LP groups (Figure 1B, C, *P* = 0.45) and no difference in MDM CD14 mRNA was observed between groups (Figure 1D) suggesting that differentiation of monocytes to macrophages was similar in LP and HP individuals. However, the percentage of CD11b^+^ cells in the MDM preparation was significantly greater in the LP group, compared with the HP group (Figure 1E, F, *P*<0.05) and the CD11b staining intensity was also higher on MDMs from the LP group, compared with the HP group (Figure 1G, *P*<0.05). Although CD11b mRNA was also enhanced in MDMs from the LP, compared with the HP, group the difference did not reach statistical significance (Figure 1H, *P* = 0.19). Since macrophages and other cells of the innate immune system recognise distinct microbial products via TLRs, we next assessed the expression of TLR2 and TLR4 since they are specifically implicated in inflammatory changes associated with neurodegeneration [14], [29]. TLR2 expression on CD11b^+^ cells was similar on PBMCs isolated from the HP and LP groups (Figure 1I, J) but expression in MDMs from the LP group was higher than the HP group, although it did not reach statistical significance (Figure 1K, L, *P* = 0.056); MFI values for the HP and LP groups were 88.1 and 77.2 respectively. TLR2 mRNA was significantly greater in MDMs prepared from the LP group compared with the HP group (*P*<0.05; Figure 1M). TLR4 expression on CD11b^+^ cells was similar on PBMCs isolated from the HP and LP groups (Figure 1N, O) but expression on MDMs from the LP group was significantly greater than from the HP group (*P*<0.05; Figure 1P, Q); MFI values for the HP and LP groups were 37.1 and 28.3 respectively. TLR4 mRNA was significantly greater in MDMs prepared from the LP group compared with the HP group (*P*<0.05; Figure 1R). Overall, this indicates that differentiated MDMs, but not freshly prepared PBMCs, isolated from the LP cohort display elevated expression of CD11b and TLR2/4.

### MDMs from the LP Group are Hypersensitive to LPS

**Figure 2:**
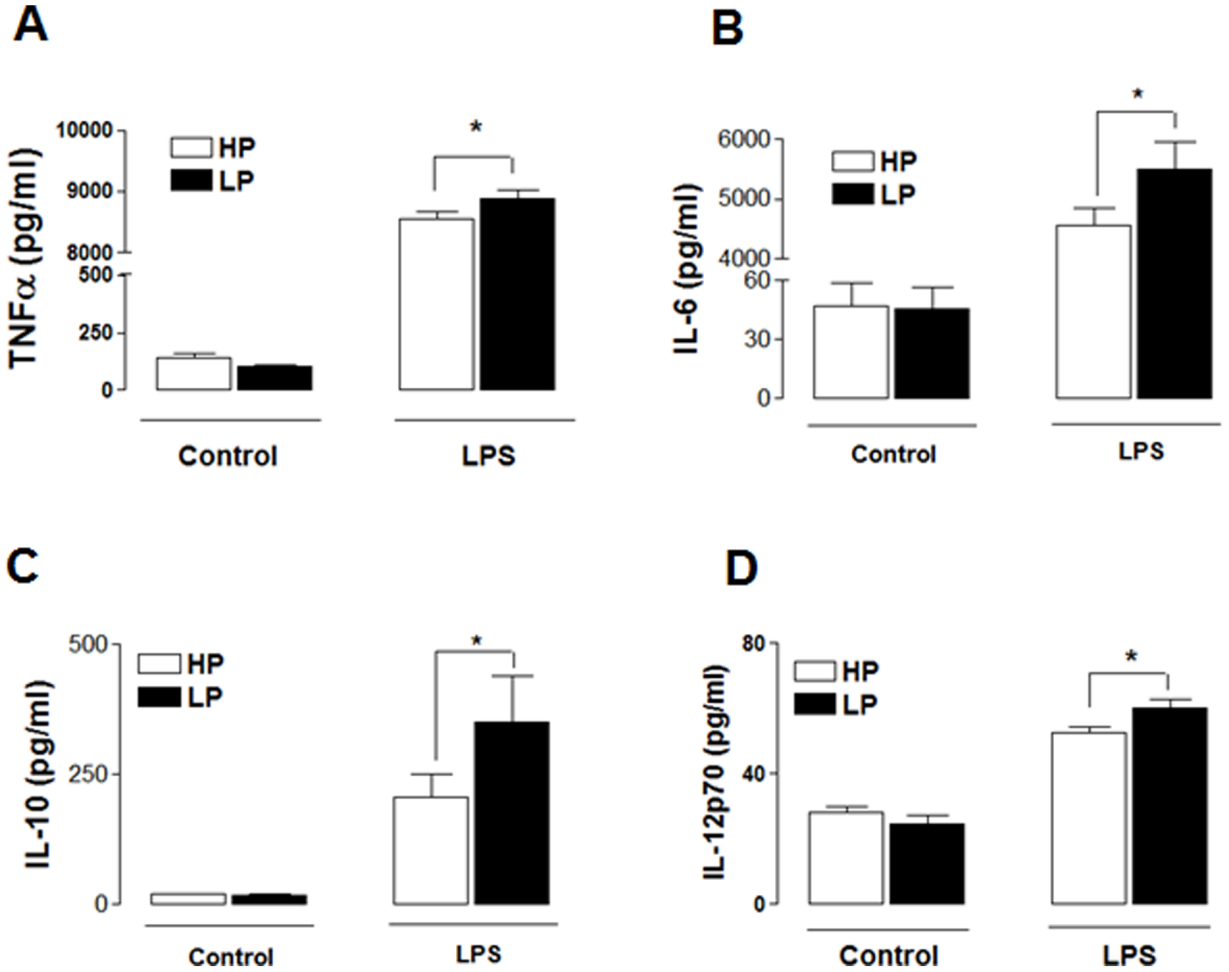
MDMs from LPs are hypersensitive to LPS. LPS (100 ng/ml; 24 h) increased A) TNFα, B) IL-6, C) IL-10 and D) IL-12p70 in MDMs prepared from HPs and LPs. The production of each cytokine was exaggerated in MDMs prepared from LPs, compared to HPs (*P*<0.05). Data are presented as mean ± SEM and represent duplicate determinations from 35 and 12 samples per HP and LP groups, respectively.

Since TLR4 acts as the primary signalling receptor for Gram-negative bacterial LPS [16] and because TLR4 expression was increased in MDMs prepared from the LP cohort, we examined the effect of LPS on cytokine production in MDMs from HP and LP groups. Production of the cytokines TNFα, IL-6, IL-10 and IL-12p70 was similar in unstimulated MDMs from HP and LP groups (Figure 2). However, LPS significantly increased cytokine production in MDMs from both groups, and importantly the production of TNFα (Figure 2A), IL-6 (Figure 2B), IL-10 (Figure 2C) and IL-12p70 (Figure 2D) was exacerbated in MDMs prepared from the LP group, compared with the HP group (*P*<0.05). This indicates that MDMs prepared from the LP group are hyper-responsive to LPS, at least in terms of cytokine production, and the increase in TLR4 expression provides a plausible mechanism for this effect.

## Discussion

We investigated the expression of TLR2 and 4, and the impact of *in vitro* treatment with LPS on the production of cytokines in MDMs prepared from an IQ memory–discrepant LP cohort, compared with an IQ memory-consistent HP cohort. The significant finding is that MDMs from the LP group expressed higher levels of TLR2 and TLR4, as well as CD11b, and exhibited a more robust response to LPS, than MDMs from the HP group.

Ageing is associated with defects in both the innate and adaptive arms of the immune system. Indeed, elderly individuals display reduced B cell production [30] and T cell memory [31], ensuring greater susceptibility than young individuals to bacterial and viral infection. Much data also indicate that ageing affects cells of the innate immune system, particularly associated with alterations in neutrophil [32] and monocyte/macrophage phagocytic capacity [33], and ability to produce cytokines and chemokines [33]. Overall, cumulative data indicates that defective innate immunity is associated with advancing age. Intermediate stages between normal ageing and dementia are recognised by several classification systems; the best recognised are the several clinical subtypes of MCI, a preclinical stage of AD [34]. The lack of diagnostic tools which enable early detection of conditions like MCI and/or AD is a major stumbling block for treatment of disease and a method of detecting early changes in cognitive function relative to previous levels would be a significant advance. Structural changes which are indicative of conversion from normal ageing to MCI and AD have been identified using image analysis [35]. In addition, a number of peripheral blood-based biomarkers have been suggested, however the most compelling data have been obtained from analysis of cerebrospinal fluid (CSF) in which the ratio of Aβ/phosphorylated tau combined with total tau levels yielded a positive predictive value in terms of conversion from MCI to AD [36].

Myeloid cells, constituting blood-borne monocytes, tissue resident macrophages and parenchymal microglia, have defined functions in neurodegenerative diseases. Monocytes and macrophages not only share the same origin with microglia in the brain, but also have the same antigens, functions, and regulatory mechanisms [37]. Much research has focused on characterising the neurodegenerative role of microglial cells which accumulate at senile plaques in AD [9], secreting proteolytic enzymes that degrade Aβ [38] and expressing receptors that promote the phagocytosis of Aβ [39]. Monocytes and macrophages are professional phagocytic cells and central players in orchestrating the innate immune response. Monocytes/macrophages have pro-inflammatory and cytotoxic properties, with the proclivity to produce inflammatory molecules such as TNFα and inducible nitric oxide synthase [37]. Blood-borne monocytes are highly mobile and rapidly recruit to inflamed CNS tissue during bacterial infection [40], autoimmune disorders [41] and neurodegenerative disease [8]. Indeed, circulating monocytes can infiltrate the CNS parenchyma and differentiate into microglia [42], where they are important for clearing amyloid and limiting its deposition in murine models of AD [43]. Furthermore, macrophages display an activated phenotype in neurodegenerative disease, typified by increased expression of cell surface markers such as CD11b [44], CNS infiltration [45] and enhanced production of inflammatory cytokines [46].

Here we show that expression of TLR4 was greater in MDMs obtained from the LP cohort compared with the HP cohort and, consistently, that incubation of cells from the LP cohort responded more robustly to LPS with significantly greater production of TNF, IL-6 and IL-12 than that produced by MDMs from the HP group. MDMs are a type I or classically activated (M1) macrophage that display a pro-inflammatory phenotype [47]. MDMs are considered a peripheral counterpart of microglia, as they share the same progenitor and antigen markers, and they have similar biological behaviours that mirror microglial function in the brain [48], [49]. Previous evidence has shown that they produce inflammatory cytokines in response to LPS treatment [50] and that they express TLR4 [51], which is confirmed here. Interestingly monocytes from older, compared with younger, individuals have higher intracellular levels of TNF both at baseline and following LPS stimulation [6], while pro-inflammatory cytokine [52] and chemokine [53], [54] levels are elevated in peripheral blood monocytes isolated from the elderly after LPS stimulation. In addition, PBMCs from AD patients have been reported to produce enhanced levels of IL-6 following LPS stimulation compared with controls [55]. These findings suggest that monocyte function is dysregulated both with age and in AD, but the present study significantly extends these observations to show that similar changes can be picked up in MDMs prepared from a group of otherwise healthy adults whose memory performance is discrepant with their estimated IQ.

CD11b is a receptor for intercellular adhesion molecule family members CD54, CD102 and CD50, enabling leukocyte adhesion and migration to mediate the inflammatory response [27]. CD11b is also a typical monocyte/macrophage marker, the expression of which is increased following activation [44]. Indeed, CD11b expression is increased in monocytes from aged individuals [6] and on microglial cells in aged animals [3]. Furthermore the number of CD11b^+^ activated microglia is increased in mouse models of AD [56] and MS [57]. The present data indicate that CD11b expression was enhanced in MDMs, but not PBMCs, from the LP cohort, indicating that these cells displayed an enhanced activated phenotype. This cell type-specific effect indicates that altering the phenotype of human monocytes *in vitro* may provide a mechanism which identifies alterations of myeloid cell function in cohorts with the most subtle cognitive changes. It is of interest to note that modifications of macrophage phenotype have been suggested in conditions where clear pathology is identified, i.e. in chronic inflammatory conditions [58], autoimmune disorders [59] and AD [60].

Human monocytes express an array of TLRs [13] and our data indicate that both CD11b^+^ PBMCs and MDMs express TLR2 and TLR4. In the present study we focused on examining expression of TLR2 and TLR4 since much evidence indicates the role of these receptors in neurodegenerative conditions. Monocytes derived from elderly individuals display defective TLR signalling, particularly TLR1/2, with further decreases in relative TLR1 and TLR4 expression determined on peripheral blood monocytes from older subjects [6], [61]. TLR2/4^−/−^ mice are protected from cognitive impairment following Aβ immunisation [29] with polymorphism in the *tlr2* and *tlr4* gene linked with AD [14]. Knockout studies suggest differential effects of TLRs on AD progression; deletion of CD14, a co-receptor for TLR2/4, reduces plaque burden in a murine AD model [62], but deletion of MyD88 enhances memory deficits [63]. Importantly, evidence indicates that Aβ binds microglial TLR2 and 4 [64] suggesting that TLRs function as members of the Aβ receptor complex. Our findings indicate that the expression of TLR2 and TLR4 is increased in MDMs from the LP group, compared with the HP group. Since increased TLR expression does not occur on monocytes in healthy elderly individuals [6], while increased expression of TLR2 and TLR4 has been reported in PBMCs from patients with late-onset AD [65], the data may suggest a role for increased TLR expression in progression from the mildest manifestation of episodic memory loss to more profound cognitive decline.

Our results identify changes in the expression of receptors CD11b, TLR2 and TLR4 on MDMs isolated from a unique cohort of IQ memory-discrepant older adults. Consistent with the increased expression of TLR4, stimulation with LPS promoted enhanced cytokine production in MDMs from this LP group, compared with the HP IQ memory-consistent group. It is important to note that bacterial and(or) viral infections are associated with reduced cognitive performance [26], but no differences in plasma concentrations of CRP were identified between the cohorts in this study. These peripheral cell changes, which are indicative of inflammation, may provide a biological substrate indicative of the earliest discernible changes in episodic memory decline which may be a precursor to more profound changes that are associated with the development of neurodegenerative disease.

## Supporting Information

Figure S1 **Flow cytometric analysis of MDMs.** Flow cytometric analysis of MDMs and selection of CD14^+^ cells. CD14^+^ monocytes were separated from PBMCs using a MACS sorter. The CD14^+^ monocytes were cultured for 7 days with GM-CSF (10 ng/ml), and stained with a CD14 antibody to determine purity by flow cytometry. Gates were made using the isotype controls. After one week in culture 90.2% of cells were CD14^+^.
(TIF)

Figure S2 **Flow cytometric analysis of PBMCs.** A) Flow cytometric analysis of PBMCs and selection of live cells. PBMC gate location determined via forward/side scatter analysis of PBMCs. B) Using a logical gating strategy, live PBMCs gated for CD11b.
(TIF)
