# Supplementary figures and images for: Identifying Early Inflammatory Changes in Monocyte-Derived Macrophages from a Population with IQ-Discrepant Episodic Memory
Source: PLoS One. 2013 May 6;8(5):e63194. doi: 10.1371/journal.pone.0063194 (PMC3646027; doi:10.1371/journal.pone.0063194)

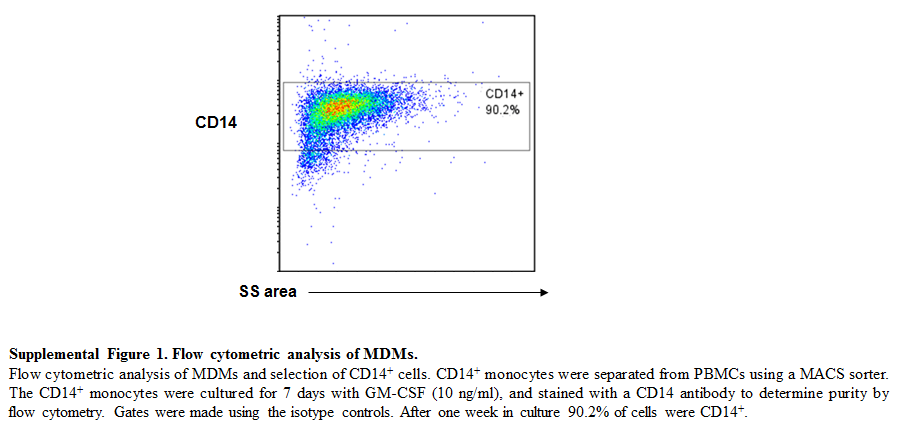

Supplement: Figure S1 — Flow cytometric analysis of MDMs. Flow cytometric analysis of MDMs and selection of CD14+ cells. CD14+ monocytes were separated from PBMCs using a MACS sorter. The CD14+ monocytes were cultured for 7 days with GM-CSF (10 ng/ml), and stained with a CD14 antibody to determine purity by flow cytometry. Gates were made using the isotype controls. After one week in culture 90.2% of cells were CD14+. (TIF) [file pone.0063194.s001.tif]

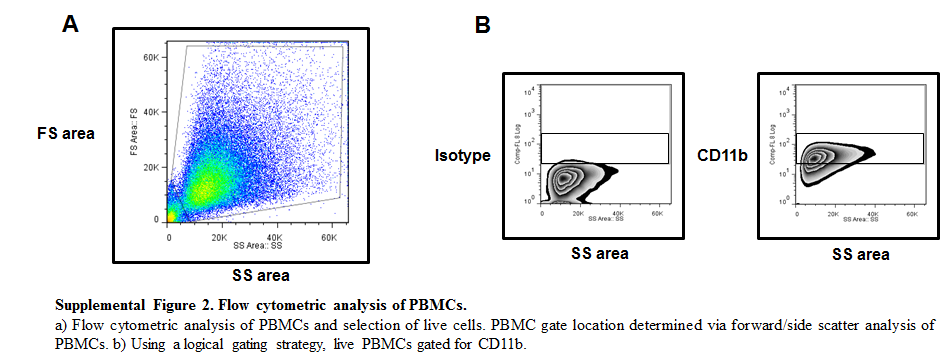

Supplement: Figure S2 — Flow cytometric analysis of PBMCs. A) Flow cytometric analysis of PBMCs and selection of live cells. PBMC gate location determined via forward/side scatter analysis of PBMCs. B) Using a logical gating strategy, live PBMCs gated for CD11b. (TIF) [file pone.0063194.s002.tif]
